# Automated Recognition of Ultrasound Cardiac Views Based on Deep Learning with Graph Constraint

**DOI:** 10.3390/diagnostics11071177

**Published:** 2021-06-29

**Authors:** Yanhua Gao, Yuan Zhu, Bo Liu, Yue Hu, Gang Yu, Youmin Guo

**Affiliations:** 1 Department of Medical Imaging, The First Affiliated Hospital of Xi’an Jiaotong University, #277 West Yanta Road, Xi’an 710061, China; gaoyanhua2012@stu.xjtu.edu.cn; 2 Department of Ultrasound, Shaanxi Provincial People’s Hospital, #256 West Youyi Road, Xi’an 710068, China; zhuyuan917@stu.xjtu.edu.cn (Y.Z.); liubo.happy@stu.xjtu.edu.cn (B.L.); 3 Department of Biomedical Engineering, School of Basic Medical Science, Central South University, #172 Tongzipo Road, Changsha 410013, China; huyuebme@stu.xjtu.edu.cn

**Keywords:** deep learning, cardiac views, graph embedding, transthoracic echocardiogram

## Abstract

In transthoracic echocardiographic (TTE) examination, it is essential to identify the cardiac views accurately. Computer-aided recognition is expected to improve the accuracy of cardiac views of the TTE examination, particularly when obtained by non-trained providers. A new method for automatic recognition of cardiac views is proposed consisting of three processes. First, a spatial transform network is performed to learn cardiac shape changes during a cardiac cycle, which reduces intra-class variability. Second, a channel attention mechanism is introduced to adaptively recalibrate channel-wise feature responses. Finally, the structured signals by the similarities among cardiac views are transformed into the graph-based image embedding, which acts as unsupervised regularization constraints to improve the generalization accuracy. The proposed method is trained and tested in 171792 cardiac images from 584 subjects. The overall accuracy of the proposed method on cardiac image classification is 99.10%, and the mean AUC is 99.36%, better than known methods. Moreover, the overall accuracy is 97.73%, and the mean AUC is 98.59% on an independent test set with 37,883 images from 100 subjects. The proposed automated recognition model achieved comparable accuracy with true cardiac views, and thus can be applied clinically to help find standard cardiac views.

## 1. Introduction

Transthoracic echocardiography (TTE) is the most commonly used cardiac imaging tool, which provides comprehensive observations of the cardiac structures and functions, and assists in the diagnosis and management of heart failure, ischemia, valve disease, and congenital abnormalities, among others [1,2]. Initially, echocardiography was a highly specialized diagnostic tool performed only by professionally trained experts, and it has now been rapidly extended to other medical specialties, especially in primary and emergency care settings [3], because it is non-invasive, cost-effective, and convenient.

However, there has been concern that the level of training of medical staff performing echocardiography in other medical specialties is not sufficient to yield accurate and reliable results. For example, incorrect quantification of left ventricular ejection fraction (LVEF) may lead to inappropriate clinical decisions [3], which may potentially harm patients and increase healthcare costs [4]. Moreover, almost all examinations in echocardiography are based on the locations of the heart views. However, the training to find standard views is time-consuming and requires expert support [5].

In order to obtain a consistent examination of echocardiography, especially in primary and emergency care settings, it is important to reduce dependence on operators [4]. Artificial intelligence is expected to provide automated analyzing tools [6].

The main challenge of ultrasound medicine is low image quality, noise, and artifacts. Because the machine learning methods based on hand-crafted or manually selected features lack robustness, deep learning based on feature learning has been applied to ultrasound image analysis in recent years [7], such as image classifications of breast cancer and benign lesions [8,9], liver cancer [10], and thyroid nodules [11]. The other applications include the quality control of fetal ultrasound and standard views of the fetus [12], and the segmentations of non-rigid organ [13] and rigid organ [14]. Three-dimensional analysis has not been widely used because of expensive calculations and limited datasets [15].

Recently, deep learning has been applied to the echocardiography in four applications [16]. The first application is evaluation of image quality in echocardiography [17]. The second application is view classification and segmentation of cardiac structures [18]. The third application is measurements: for example, quantification of left ventricular size and function [19]. The final application is detection of abnormalities such as wall motion abnormalities [20], and assessments of heart failure with preserved ejection fraction [21] and diagnosis of myocardial disease [19].

The classification of cardiac views can be useful for automated detection of appropriate views in TTE. For example, effective standard view recognition can remind less skilled operators to determine whether the obtained view is a standard view. They will get a message while finding a standard view.

Some studies have reported good classification of cardiac views with an accuracy of 84–98%. Zhang et al. trained a convolutional neural network (CNN) with multiple tasks including view classification, and the overall accuracy on 23 viewpoints was 84% [19]. Madani proposed a fast and accurate cardiac view recognition method for 15 views and doppler images, which achieved an overall accuracy of 91.7% (image classification) and 97.8% (video classification) [22]. Kusunose et al. reported the newly developed CNN for classification of cardiac views, and the overall accuracy was up to 98.1%, which was acceptable for a feasible identification model in clinical practice. However, CNN only predicts the video classes of five cardiac views [23].

The challenge comes from large intra-class differences and small inter-class differences in cardiac views. Some individual factors, such as gender, race, age, and heart diseases, may result in alterations of the same cardiac view. The cardiac surface changes periodically and non-linearly during cardiac cycles, and the shapes of some views are relatively similar, which further increases the difficulty of recognition. Echocardiographers may not be able to identify deformed cardiac views accurately enough. See Supplementary Figure S1 for nine standard cardiac views in TTE examination, including parasternal long-axis (PSLA), parasternal short-axis at the level of great vessels (SB), parasternal short-axis at the level of papillary muscles or mitral (SM), apical four-chamber view (A4C), apical five-chamber view (A5C), apical two-chamber view (A2C), apical three-chamber view (A3C), subcostal four-chamber view (SUB4C), suprasternal notch aortic arch (SUPAO).

This paper proposes an automatic recognition method to identify nine standard cardiac views. The presented method is based on CNN, which includes three effective strategies, i.e., graph regularization learning (GRL) [24,25], spatial transform networks (STM) [26], and channel attention mechanism (squeeze-and-excitation network, SE) [27]. The highlights are given as follows:

(1) The STM is performed as an independent pre-processing module, which learns the deformation during the cardiac cycle to reduce the intra-class variability. Second, the SE recalibrates channel-wise responses to enhance the features related to the recognition.

(2) The similarity between the samples is ignored in conventional deep learning. In the presented method, the structural signals of the sample similarity are defined as the graph-based embedding, which acts as an unsupervised regularization constraint to achieve accurate classification better than known methods.

## 2. Materials and Methods

### 2.1. Datasets

All the cardiac images came from two hospitals, Shaanxi Provincial People’s Hospital (SXPPH) and the First Affiliated Hospital of Xi’an Jiaotong University (XJTUFAH). 584 subjects from SXPPH and 100 subjects from XJTUFAH respectively underwent TTE examination, while four experienced echocardiographers recorded the examination videos of nine cardiac views. Their demographics were described in the Supplementary Tables S1 and S2. The number of subjects with LVEF <55% and ≥55% in XJTUFAH is equal. The experimental design was approved by the Institutional Review Board (IRB), and all subjects were informed of the experimental contents and risks.

### 2.2. Preprocessing Pipeline

In order to remove the patients’ identifiable information, the surrounding pixels of each video were cut out. A frame image was extracted from the videos at an interval of 5 frames. All images were reviewed independently by two experienced echocardiographers, while the low-quality and incorrect images were excluded, and only the images agreed by both echocardiographers were retained. Approximately 100–400 images were obtained from each subject. Finally, 171,792 (SXPPH, Dataset 1) and 37,883 (XJTUFAH, Dataset 2) images were obtained. The distribution of cardiac views is shown in Table 1 and Table 2.

The images in Dataset 1 were divided into a training, validation, and test set according to the ratio of 7:1:2. The images from the same subject were not divided into different sets for data independence. Dataset 2 was used as an independent test set to confirm generalization accuracy between different hospitals. All images were scaled to 512 × 512 pixels and converted to red, green, and blue channels for the following network framework.

### 2.3. Network Framework

**Figure 1:**
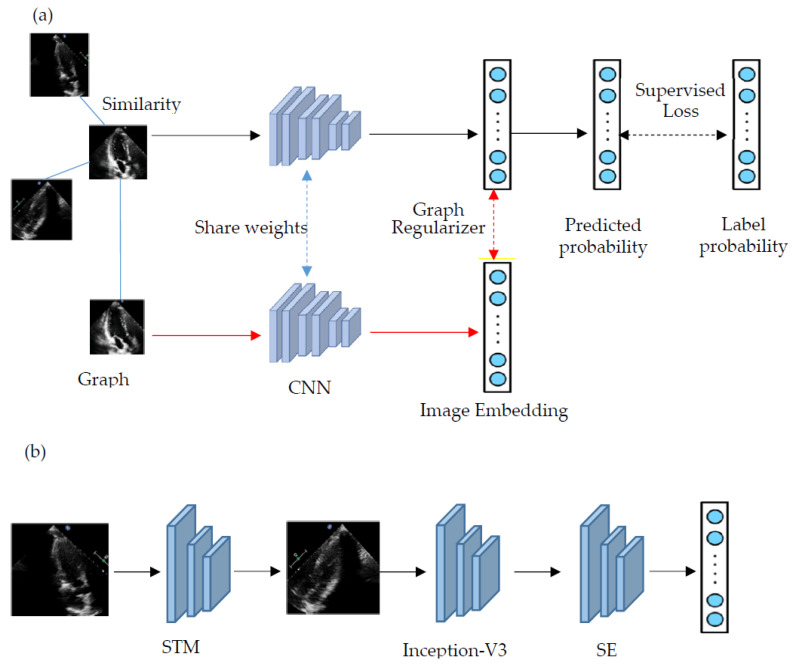
An illustration of the proposed classification framework. (**a**) The sample with similar samples defined by the similar graph are inputted into the same CNN, and the representation produced by similar samples is used as graph regularizer to compute unsupervised loss, which are combined with supervised loss to train the CNN. (**b**) The CNN includes three modules. STM learns the six affine parameters of the cardiac deformations to reduce the geometric distortion. The Inception-V3 with output layer removed is used for feature extraction. The extracted feature maps are inputted to the SE, which adaptively recalibrates channel-wise feature responses, and then the predicted probability is outputted.

A graph-constrained CNN is proposed. The graph is built based on the similarity among images. Each node on the graph represents a training image, and the edge between two nodes indicates the similarity between two images represented by the two nodes. The learning strategy is based on the assumption that more similar images are more likely to be the same labels. When an image is inputted into a neural network, the images of its adjacent nodes are also inputted in the same batch. The image embedding of adjacent nodes could be used as a graph regularizer or unsupervised graph loss, which minimizes the high-level feature difference between adjacent nodes and the inputted image. Meanwhile, the cross-entropy of the label and the predicted probability of the inputted image are then calculated as the supervised loss, as shown in Figure 1. The overall training goal is to minimize the weighted sum of supervised loss and unsupervised graph loss.

The black flow represents the conventional CNN training, and the red flow indicates that the adjacent images are inputted in the same batch (Figure 1). The inputted image and its adjacent images share the weights of the CNN.

Total loss is given as follows:

(1) $$ℛ\left(\theta \right)=ℒ\left(\theta \right)+\alpha \underset{\left(u,v\right)\in ℰ}{\sum }w_{u,v}d\left(\phi \left(x_{u}\right),\phi \left(x_{v}\right)\right)$$

ℛ(θ) represents total loss, θ represents the weights of CNN. The first term ℒ(θ) is supervised loss, and the second term is the graph regularizer. $x_{u}$ is an inputted image. $w_{u,v}$ represents the similarity between $x_{u}$ and its adjacent node $x_{v}$, which is also the edge weight between them. ϕ represents the image representation or embedding extracted from the embedding layer, i.e., highest-level feature. d is the mean square errors of the two image embeddings.

**Figure 2:**
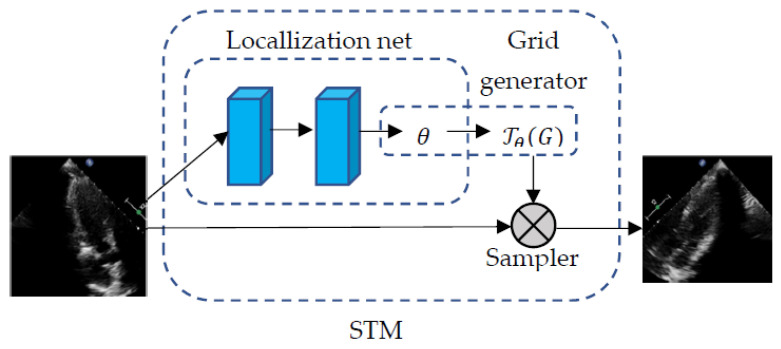
The flowchart of spatial transformer network. STM receives original images and uses a localization network to learn the parameters of the geometric transformation during the cardiac cycle. This network consists of two layers. First, the convolution layer of 5 × 5 filters is used to output 128 feature maps, followed by a global averaging layer and a fully connected layer. The localization network produces 6 parameters of affine transformation. The transformation parameters are in turn applied to every inputted image to perform a geometric transformation and reduce cardiac deformations.

**Figure 3:**
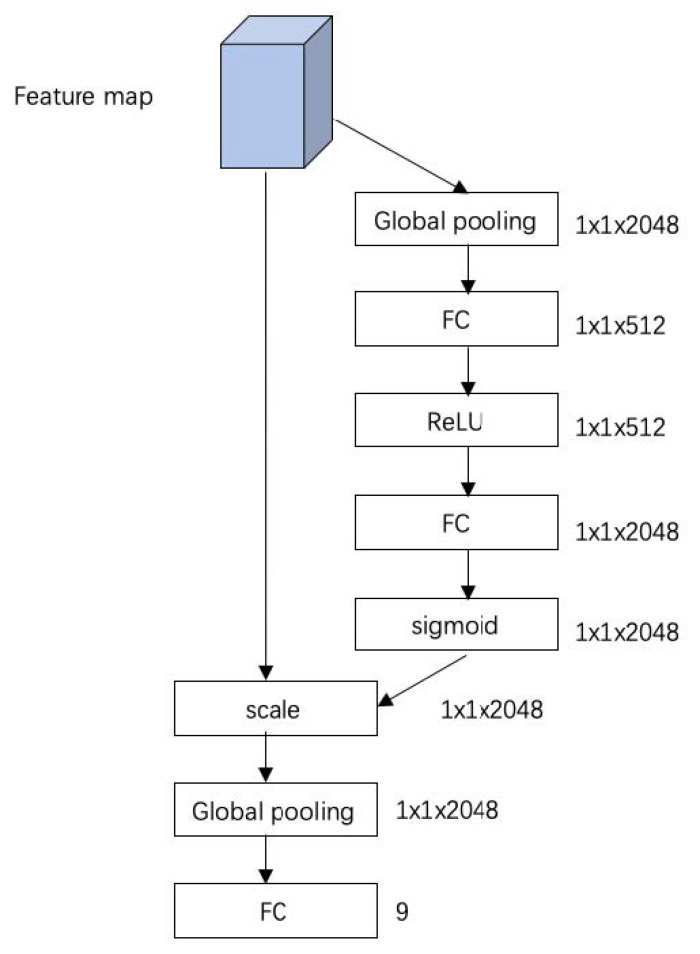
The flowchart of SE. As a feature extractor, Inception-V3 is removed from the final output layer, and the outputted feature maps with 2048 channels are fed into SE, where the channel attention mechanism is introduced to enhance the channel that are more effective for accurate predictions.

Our CNN consists of three functional modules, namely the STM (Figure 2). Inception-V3 [28] and SE (Figure 3). Inception V3 is a famous CNN and has achieved excellent performance in many image classifications. Although Inception V3 has shown some translation invariance, it cannot handle the deformations during the cardiac cycle phase well. The STM uses a localization network to learn the parameters of the geometric transformation of heart motion, and the transformed image is then inputted to the Inception-V3. The feature maps with 2048 channels from the highest-level layer of Inception-V3 are then inputted into the SE. SE introduces the channel attention mechanism, which enhances the channel of feature maps that are more effective for accurate predictions.

### 2.4. Graph Construction

During the same cardiac cycle, the ultrasound images are always changing, but they are similar. We used mutual information of two images to represent their similarity. Firstly, for any image in the training set, it is added to the graph as a node. Secondly, the mutual information of every node and another node/image from the same category is calculated. If the mutual information of the two nodes is greater than a threshold, the two nodes are connected, that is, there is an edge between the two nodes. Thirdly, the mutual information is used as their edge weights. Fourthly, in order to control the graph size, the threshold is set to 0.2, and each node has at most 10 neighbor nodes with the greatest similarity.

### 2.5. Training Process

The hyper-parameters are selected based on the validation set. The combinations of some hyper-parameters such as learning rate, batch size, training epoch are tested, and the parameters with highest accuracy on the validation set are used. The network is implemented on the software Python (version 3.6.9) [29] and Tensorflow (version 1.15.0) [30], on the server with a NVIDIA Tesla V100 Graphic Processing Unit (GPU), 128 GB memory, and two Intel Xeon Gold 5122 Central Processing Units.

The weights of STM and SE are initialized with the glorot uniform initializer. The Inception V3 is initialized by the pre-trained weights on ImageNet dataset and the deep fine-tuning based on ultrasound images is then performed. Because the image and the adjacent nodes are loaded together in a batch, the number of images is limited by the GPU memory. For V100 GPU, the batch size can be set to be 32, where the number of inputted images is 8, and the 4 adjacent images of each inputted image are randomly selected. Based on the hyper-parameter selection, the optimizer is set to Adam, the training epoch is 500, with 100 steps in each epoch. The learning rate is initialized to 0.0001. When the validation accuracy does not improve for 5 epochs, the training is stopped. We compared the performance of α in Formula (1), and found α=0.4 can get the best accuracy. During training, we do not use data augmentation, because the calculation of the graph would be greatly increased. The hyper-parameter sets refer to Table 3.

To illustrate the role of STM, Graph and SE, we also combine these three modules, Inception V3, Inception V3 + SE, STM + Inception V3 + SE separately and compare them with the proposed method, Graph + STM + Inception V3 + SE. While the training of Inception V3, Inception V3 + SE, STM + Inception V3 + SE, the batch size is set to 32, and data augmentation is used, such as random flips, rotations, etc., the optimizer, training epoch, and learning rate are the same as those in Table 3.

The metrics, such as accuracy, sensitivity, specificity, and AUC (Area under curve), are used for performance evaluation. All metrics are calculated separately in a single category (cardiac view), defining the current category as positive class, and the other 8 categories are defined as negative classes. The accuracy is defined as the number of correctly classified samples divided by the number of all samples. The sensitivity is defined as the number of correctly classified positive samples divided by the number of all positive samples. The specificity is defined as the correctly classified negative samples divided by all the negative samples. The AUC describes overall performance of sensitivity and specificity. Finally, the overall accuracy was calculated over all 9 categories. Moreover, the confusion matrix shows the misclassification among the nine categories. The t-SNE cluster [31] and occlusion experiment further confirm the performance.

## 3. Results

The datasets from two hospitals include 171,792 images (Table 1) and 37,883 (Table 2). Firstly, the training and testing are performed on the Dataset 1, and then independent tests are performed on the Dataset 2.

The accuracy of Inception V3 is 88.78%. After channel attention is introduced, the overall accuracy of Inception V3 + SE improves significantly. STM reduces the variability of cardiac deformation, and slightly improves the accuracy to 96.50%. Because the graph regularization serves as a robust unsupervised loss, the proposed method achieves the best overall accuracy of 99.10% (Table 4).

The evaluation on cardiac views is shown in Table 5. PSLA, SB, SM, SUB4C and SUPAO are all recognized, with the sensitivity of 100%, and no images are misclassified into other categories, and the AUC reaches 100%. The A5C, A2C, and A3C are slightly misclassified. In particular, the sensitivity of A2C is 94.63%.

The evaluation on independent test set is shown in Table 6. The SM, SB, SUB4C are all correctly identified. However, few images of the PSLA, SUPAO are mistakenly classified. Similarly, some images of A4C, A5C, A2C, A3C are easily confused. In particular, the sensitivity of A2C is reduced to 94.15%. The overall accuracy of each category is all higher than 97%, and mean AUC is more than 98%, although the results in Table 6 are only slightly worse than those of Table 5.

**Figure 4:**
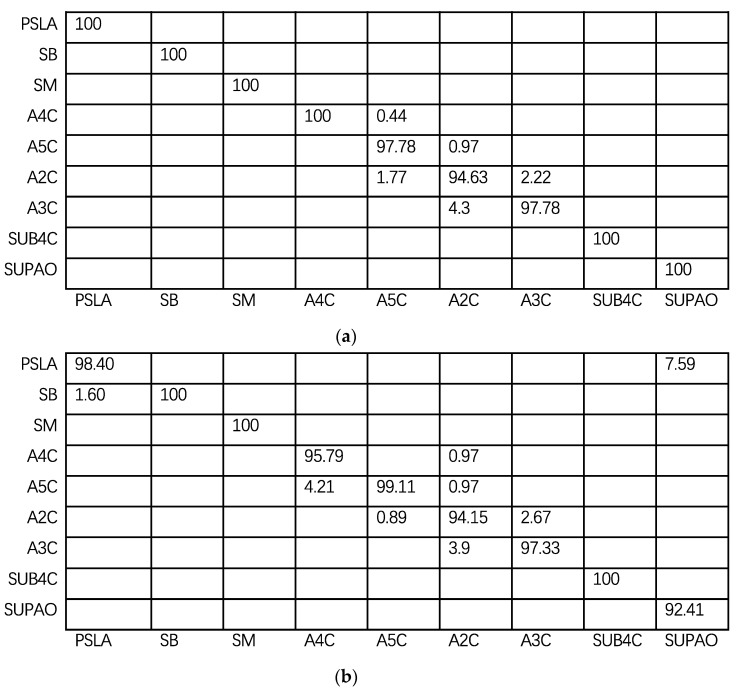
Confusion matrices. (**a**) Testing set in Dataset 1, (**b**) Testing set in Dataset 2.

In order to find the classification errors among cardiac views, the confusion matrices are computed. As shown in Figure 4, the horizontal axis is the true labels, and the vertical axis is the predicted labels. The numbers in Figure 4 are the percentages of predicted labels. On the diagonal, the closer the number is to 100, the more accurate the predicted labels are.

Figure 4a shows the confusion matrix of the test set in Dataset 1; Figure 4b is the confusion matrix in Dataset 2. The classification of SB, SM, SUB4C are accurate enough. However, the misclassification mainly occurs among A4C, A5C, A2C and A3C. In particular, A2C and A3C are easily confused. In Figure 4b, about 3.9% of A2C is misclassified as A3C, and the 2.67% of A3C is misclassified as A2C.

In Supplementary Figure S2, after deep learning, the SB, SM, SUB4C are completely distinguishable. Only a few samples of A4C, A5C, A2C and A3C are mixed together, which is consistent with the confusion matrices. In Supplementary Figure S3 and Table S3, our method can find important heart tissues (obscured areas) in images.

## 4. Discussion

TTE is one of the most important cardiac examinations because it is non-invasive, cost-effective, convenient. The accuracy and reproducibility of TTE rely on the accurate recognition of cardiac views. However, the recognition depends on echocardiographers’ experiences, and implementation of artificial intelligence is expected to provide a good solution.

The datasets came from nearly 700 patients from two hospitals. The four echocardiographers had excellent skills on TTE, and they recorded all videos of cardiac views. In order to ensure the independence of subsequent study, two other echocardiographers reviewed all the images, excluding some unqualified images.

The main challenges of recognition for cardiac views are low-quality images and shape changes during the cardiac cycle. The Inception V3 is one of the most commonly used networks for image recognition, but its overall accuracy is only 88.78%. Because the number of outputted channels by Inception V3 becomes 2048, the explicitly modeling interdependencies between channels can be expected to improve the performance across multiple datasets and tasks [27]. After recalibration of channel-wise feature responses is introduced by SE, the recognition becomes more effective through channel attentions. STM is also useful because it models the geometric deformation of cardiac views by affine transform, which reduces the impact of the cardiac cycle on the recognition effectively. The accuracy is increased to 96.5%. To our knowledge, this result is better than the previous results [23].

Unlike conventional deep learning, the structural signals are introduced by the similarity between samples to learn relationships among them. Ideally, the graph regularization can reduce the amount of labeled data and generalization errors. The first step of graph regularization is to build a graph. In general, the similarity between two images is not easy to evaluate based on pixel-level comparisons. The cardiac images in the same cardiac cycle are similar and appear periodically. Therefore, the mutual information between two images can be used as a measurement of the edge weights. We introduced graph regularization to STM-Inception V3-SE network, which further improved the accuracy by about 2%.

Nine usual cardiac views are studied for automatic recognition. The overall accuracy of the four networks are tested, and it is confirmed that the presented method achieves the best accuracy of 99.10%. The sensitivity, specificity, accuracy, and AUC values are also calculated for each of the nine categories, respectively. The recognition of PSLA, SB, SM, SUB4C and SUPAO show good performances, with a sensitivity of 100% and an AUC of more than 99%. A4C, A5C, A2C and A3C are slightly misclassified among them, but the mean AUC is higher than 98%.

The confusion matrices analysis further confirm the above results. In particular, the A2C and A3C are not easily classified. This result indicated the next improvement direction, especially for A2C. Moreover, the overall accuracy on independent test set is 97.73%. The proposed method could be generalized to new datasets.

The comparison of our method and other recent methods is shown in Table 7 including the number of test set, accuracy and AUC. Zhang et al. [19] designs a full automated method, but their overall accuracy of view classification is only 84%. Madanis’ method achieves 91.7% accuracy on 15 kinds of still images including cardiac views and doppler images, which is not satisfactory for clinical application. Although Kusonose et al. report a better method, where the overall accuracy in an independent test set is 98.1%, but the number of cardiac views is only 5. Moreover, the accuracy is based on the average of 10 selected images of video classification. In contrast, our method has an accuracy of 97.73% on nine kinds of cardiac views or images.

It is worth noting that the proposed method is based on the datasets of standard views. In clinical practice, the classification of standard views can be used as an assisted tool. For example, if the view obtained by the operator cannot be recognized as one of standard views, then this view should be non-standard, which will help less skilled operators to find more accurate views. Because the recognition or evaluation of non-standard views is also valuable, a large number of non-standard views will be included for model training in future studies.

The main contribution is to propose an effective method for the recognition of standard cardiac views. As far as we know, the obtained results are the most accurate. Because our dataset is not large enough, we believe this accuracy will be further improved by more training data. Moreover, in order to confirm the feasibility of deep learning on echocardiography, more data from other hospitals including non-standard views should be used for testing.

## 5. Conclusions

This paper proposed an effective CNN method for identifying cardiac views. Three modules are introduced to reduce shape deformation caused by the cardiac cycle, recalibrate channel-wise feature responses, and improve the accuracy by graph constraint. The evaluation of two datasets has shown the high performance of the proposed method, which is expected to be an assisted tool for detection of appropriate standard views in TTE.

## Figures and Tables

**Table 1:** Distribution of cardiac views from SXPPH.

Cardiac Views	PSLA	SB	SM	A4C	A5C	A2C	A3C	SUB4C	SUPAO	Total
Number	27,888	25,355	28,546	15,433	18,270	14,760	17,099	12,988	11,455	171,792

**Table 2:** Distribution of cardiac views from XJTUFAH.

Cardiac Views	PSLA	SB	SM	A4C	A5C	A2C	A3C	SUB4C	SUPAO	Total
Number	3763	5100	4582	3798	4502	4098	4499	4381	3160	37,883

**Table 3:** The hyper-parameter sets.

Hyper-Parameter	Value
Optimizer	Adam
Train epoch	500
Steps per epoch	100
Learning rate	0.0001
Alpha	0.4
Batch size	8
Adjacent number	4
Dropout rate	0.5

**Table 4:** The overall accuracy of four models in Dataset 1.

Networks	Overall Accuracy
Inception V3	88.78%
Inception V3 + SE	94.65%
STM + Inception V3 + SE	96.50%
Graph + STM + Inception V3 + SE ^a^	99.10%

^a^: presented method.

**Table 5:** Test results of the presented method on Dataset 1.

Cardiac View	Sensitivity	Specificity	Accuracy	AUC
PSLA	100%	100%	100%	100%
SB	100%	100%	100%	100%
SM	100%	100%	100%	100%
A4C	100%	99.82%	99.84%	99.91%
A5C	97.78%	100%	99.74%	98.89%
A2C	94.63%	99.47%	98.94%	97.05%
A3C	97.78%	99.16%	99.00%	98.47%
SUB4C	100%	100%	100%	100%
SUPAO	100%	100%	100%	100%
Total views	-	-	99.10% ^a^	99.36% ^b^

^a^: overall accuracy; ^b^: mean AUC.

**Table 6:** Test results of the presented method on Dataset 2.

Cardiac View	Sensitivity	Specificity	Accuracy	AUC
PSLA	98.40%	99.30%	99.21%	98.85%
SB	100%	99.82%	99.84%	99.91%
SM	100%	100%	100%	100%
A4C	95.79%	99.88%	99.47%	97.84%
A5C	99.11%	99.40%	99.37%	99.26%
A2C	94.15%	99.52%	98.94%	96.84%
A3C	97.33%	99.53%	99.26%	98.43%
SUB4C	100%	100%	100%	100%
SUPAO	92.41%	100%	99.37%	96.20%
Total views	-	-	97.73% ^a^	98.59% ^b^

^a^: overall accuracy; ^b^: mean AUC.

**Table 7:** The comparison of our method and other recent methods.

Networks	Year	Test Set			Independent Test Set		
		Images/Subjects	Accuracy	AUC	Images/Subjects	Accuracy	AUC
Zhang et al. [19]	2018	14,035/277	84%	-	-	-	-
Madani et al. [22]	2018	20,000/27	91.7%	-	-	-	-
Kusunose et al. [23]	2020	-	-	-	1890/189	98.1% ^a^	-
Our method		34,358/117	99.10%	99.36%	37,883/100	97.73%	98.59%

^a^: accuracy of video classification.

## Data Availability

The data presented in this study are available on request from the corresponding author. The data are not publicly available due to [the protecting provision about the patients’ privacy included in informed consent].

## Supplementary Materials

The following are available online at https://www.mdpi.com/article/10.3390/diagnostics11071177/s1, Figure S1: Ultrasound images of nine standard cardiac views during cardiac cycles, Table S1: clinical information of Dataset 1 from SXPPH. Table S2: Clinical information of Dataset 2 from XJTUFAH. Figure S2: Visualization analysis based on t-SNE clusters. Figure S3: The result of occlusion experiment.
