# Supplementary material for: Automated Recognition of Ultrasound Cardiac Views Based on Deep Learning with Graph Constraint
Source: Diagnostics (Basel). 2021 Jun 29;11(7):1177. doi: 10.3390/diagnostics11071177 (PMC8303427; doi:10.3390/diagnostics11071177)
Supplement: Supplementary file 1 [file diagnostics-11-01177-s001.zip › diagnostics-1250381-supplementary.pdf]

Supplementary materials

(1) Supplementary Figure S1

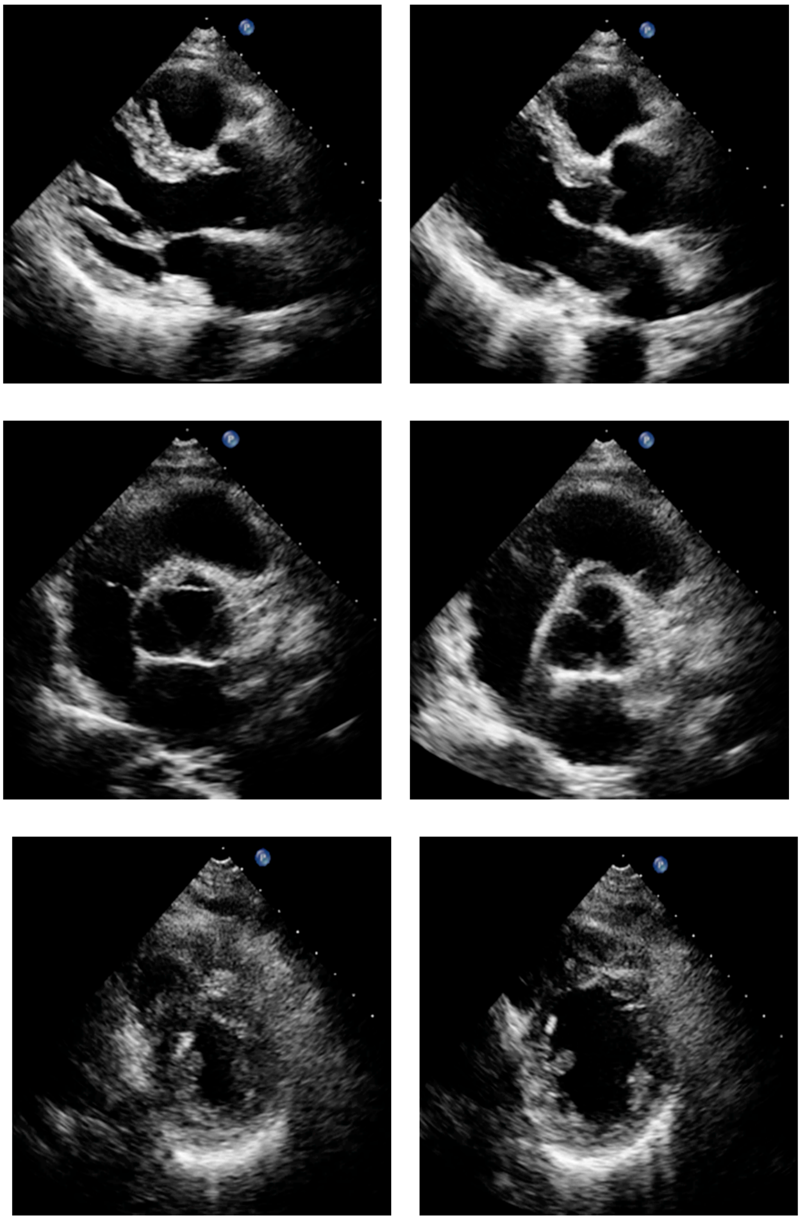

PSLA

SB

SM

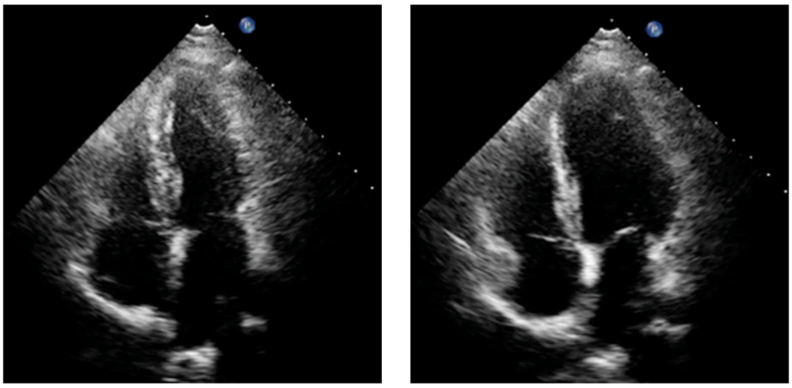

A4C

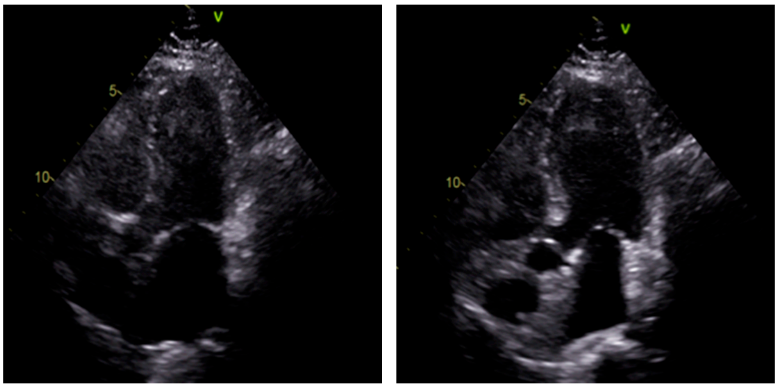

A5C

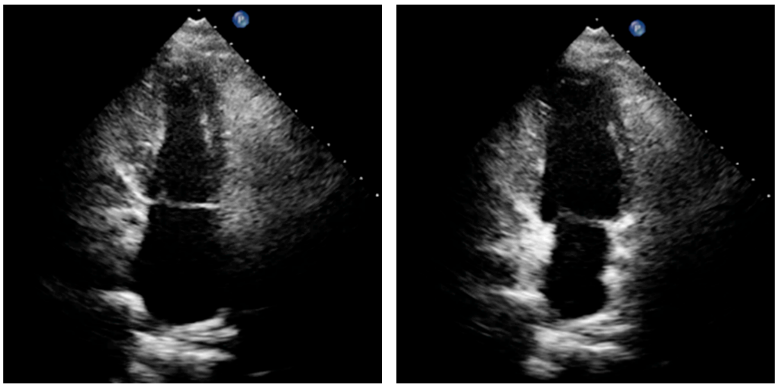

A2C

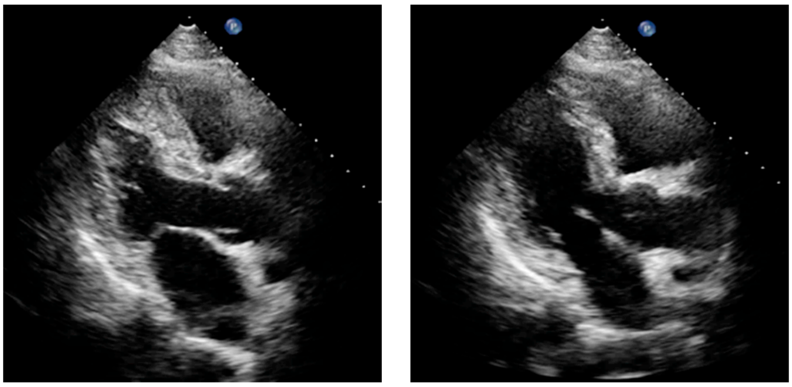

A3C

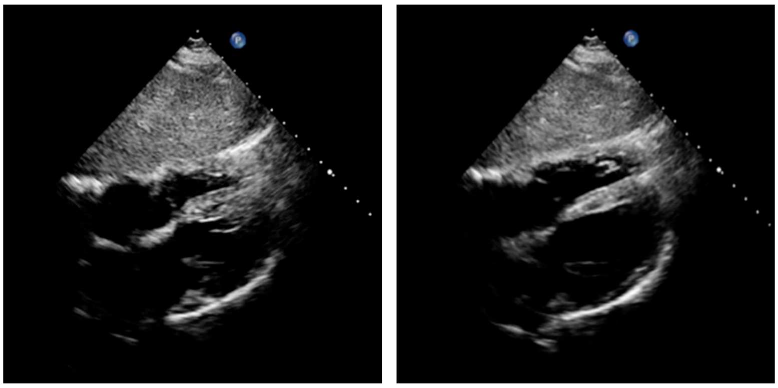

SUB4C

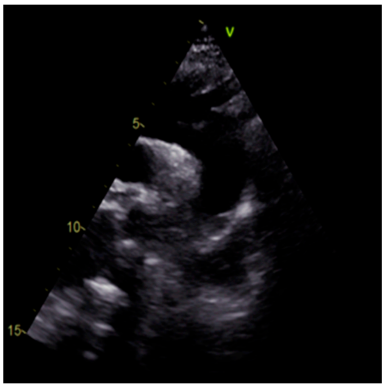

SUPAO

Figure. S1. Ultrasound images of nine standard cardiac views during cardiac cycles. There are 2 images in each row, the left is the image in systole and the right is in diastole.

(2) Supplementary Table S1 and S2：clinical information of two datasets

All the subjects came from two hospitals, SXPPH and XJTUFAH. While TTE examination, the gender, age and clinical indications were also recorded, as shown in supplementary Table S1 and supplementary Table S2, but the subjects' identity information was removed.

Table S1. Clinical information of Dataset 1 from SXPPH

Patients Demographics	
Number of patients	584
Age, mean±SD[a], years	53±13
Age range (years)	25-83
Male gender, number (percentage)	319(54.6%)
BMI (kg/m^2)	22.5±3.5
Pathology, number (percentage)	
LVEF<55%	38(6.5%)
RVSP>40	27(4.6%)
Normal studies[b]	75(12.9%)
2D-Echocardiographic findings	
Ao-s(d), mean±SD, (mm)	30±3.7
Ao-asc(d), mean±SD, (mm)	30±3.9
MPA(d), mean±SD, (mm)	21±2
LA-ap(d), mean±SD, (mm)	33±5.3
LA-t(d), mean±SD, (mm)	34±5.6
RA-t(d), mean±SD, (mm)	32±3.6
RV-ap(d), mean±SD, (mm)	23±3.9
RV-t(d), mean±SD, (mm)	25±2.6
LVEDD, mean±SD, (mm)	48±5.9
LVESD, mean±SD, (mm)	33±6.1
IVSd, mean±SD, (mm)	9.5±1.1
LVPWd, mean±SD, (mm)	9.5±1.0

Supplementary Table S2. Clinical information of Dataset 2 from XJTUFAH

Patients Demographics	
Number of patients	100
Age, mean±SD[a], years	58±12
Age range (years)	22-80
Male gender, number (percentage)	58(58%)
BMI (kg/m2)	24.7±2.8
Pathology, number (percentage)	
LVEF<55%	50(50.0%)
RVSP>40	13(13.0%)
Normal studies[b]	7(7.0%)

2D-Echocardiographic findings	
Ao-s(d), mean±SD, (mm)	33±5.1
Ao-asc(d), mean±SD, (mm)	33±5.7
MPA(d), mean±SD, (mm)	22±3.4
LA-ap(d), mean±SD, (mm)	38±8.7
LA-t(d), mean±SD, (mm)	40±10.2
RA-t(d), mean±SD, (mm)	32±6.5
RV-ap(d), mean±SD, (mm)	22±3.4
RV-t(d), mean±SD, (mm)	25±3.7
LVEDD, mean±SD, (mm)	72±8.4
LVESD, mean±SD, (mm)	62±9.9
IVSd, mean±SD, (mm)	9.9±2.4
LVPWd, mean±SD, (mm)	9.6±1.5

Ao-s(d): Aortic sinus diastolic diameter, Ao-asc(d): ascending aortic diastolic diameter, MPA(d): main pulmonary artery diastolic diameter， LA-ap(d): Left atrial anteroposterior diastolic diameter， LA-t(d): Left atrial transverse diastolic diameter, RA-t(d): Right atrial transverse diastolic diameter, RV-ap(d): Right ventricular anteroposterior diastolic diameter, RV-t: Right ventricular transverse diastolic diameter, LVEDD: Left ventricular end diastolic diameter, LVESD: Left ventricular end systolic diameter， IVSd: Ventricular septal diastolic thickness, LVPWd: Left ventricular posterior wall diastolic thickness

a: mean±SD: average value±standard deviations

b: Defined by echo reports documenting normal four-chamber size and systolic/diastolic function, normal valves with trace or less regurgitation, no wall motion abnormalities, normal great vessels and estimated right atrial pressure, no pericardial effusion, and no other abnormalities.

（3）Supplementary Figure S2: Visualization analysis based on t-SNE clusters

Deep learning can extract high-dimensional representations, and the performance of the classifier can be shown based on the separability on high-dimensional space. The various types of clusters before and after learning can be shown by t-SNE cluster analysis. The left image is the t-SNE clusters of original images, where each image is represented as a 512 * 512-dimensional high-dimensional vector, and then projected into a two-dimensional vector for visualization purposes. We used different color points to represent different classes of cardiac views. The original images are not clear clusters, and the images cannot be distinguished from the original image space. The right image is the high-dimensional representation extracted by our method. The output before last fully connected layer that is the 2048-dimensional vector, which is projected into the 2-dimensional space by t-SNE, showing clearly category-based clusters.

In order to reduce the amount of data, the colors in ultrasound images are removed from red, green, blue channels to gray channel, which is flattened into 512 * 512-dimensional vector. t-SNE is initialized with principal component analysis (PCA).

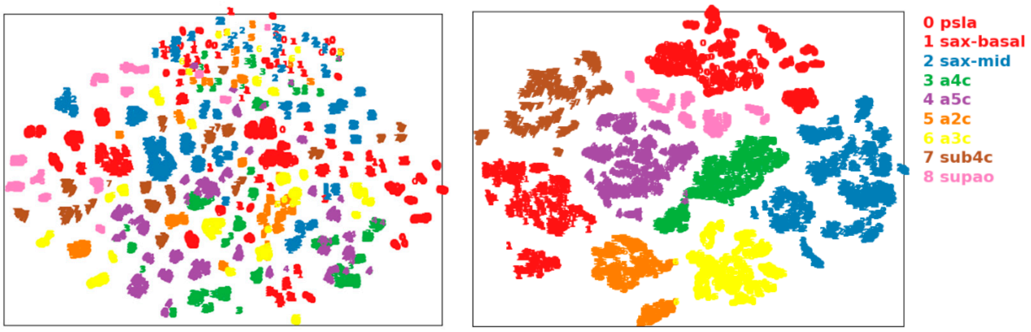

(a) (b)

Supplementary Figure S2. Visualization analysis of t-SNE clustering before and after deep learning. Figure (a) is the t-SNE results of the original image. (b) is the t-SNE results after deep learning.

（4）Supplementary Figure S3. The result of occlusion experiment

For a test image, an ultrasound doctor occluded some region, and the predicted probability is given by the proposed method. In Supplementary Figure S4, a typical image was selected for each category, including the original image, partially occluded image and completed occluded image. the predicted probabilities are shown in Table 8.

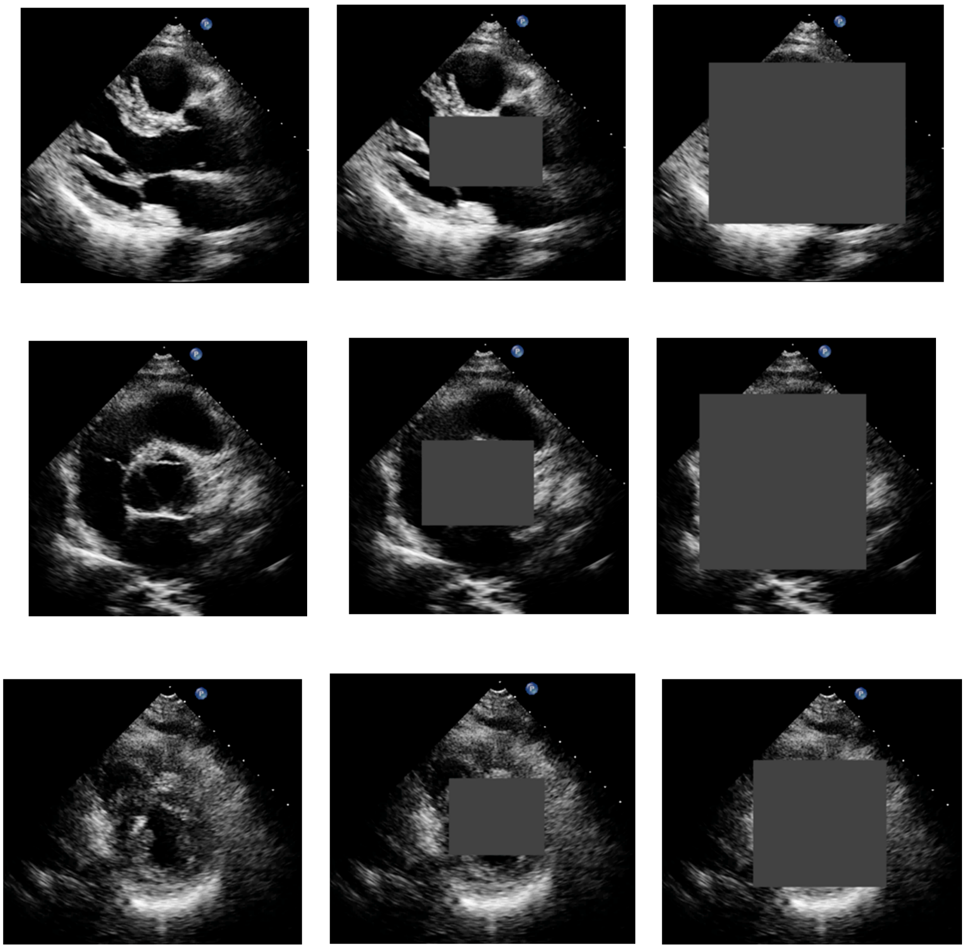

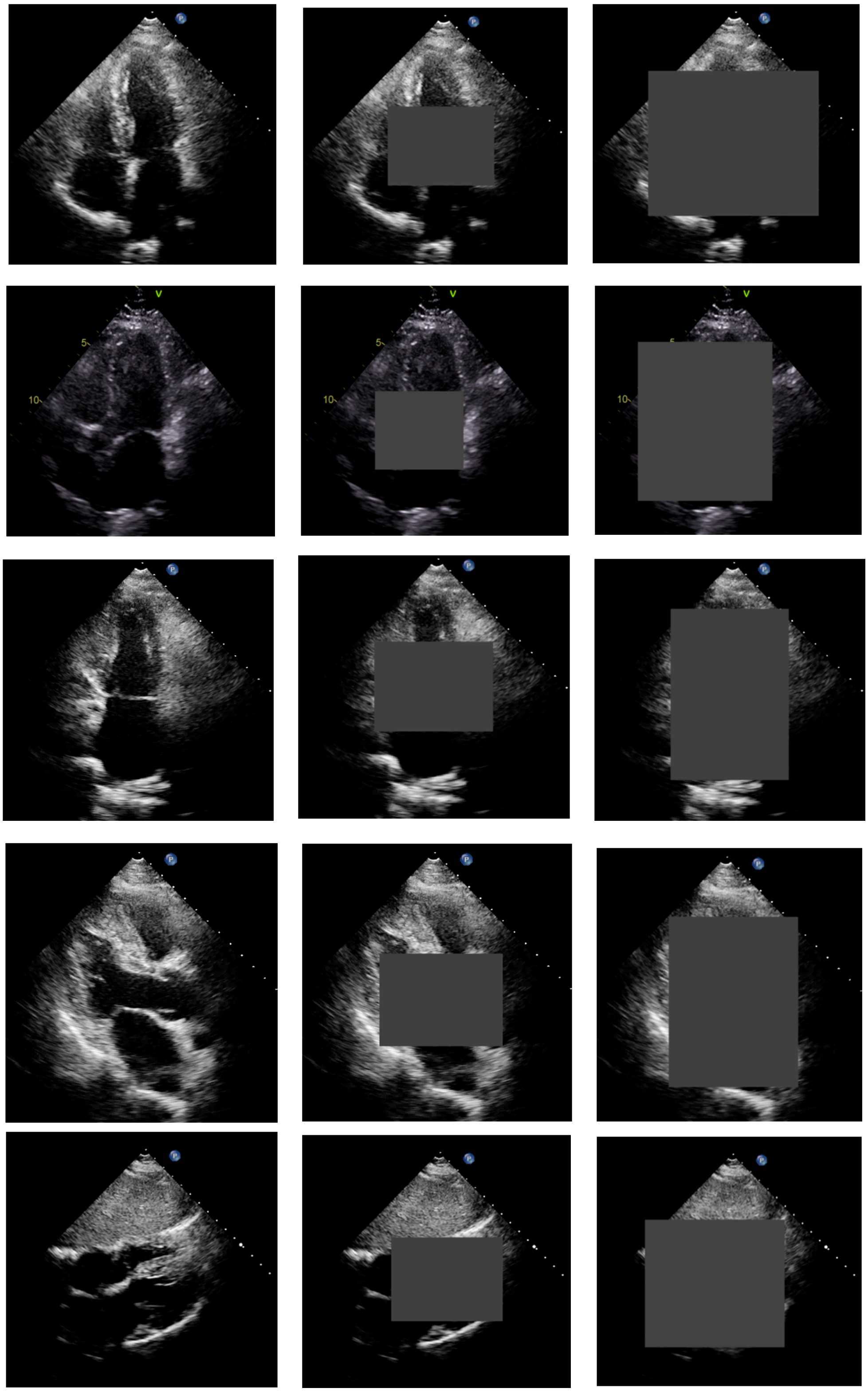

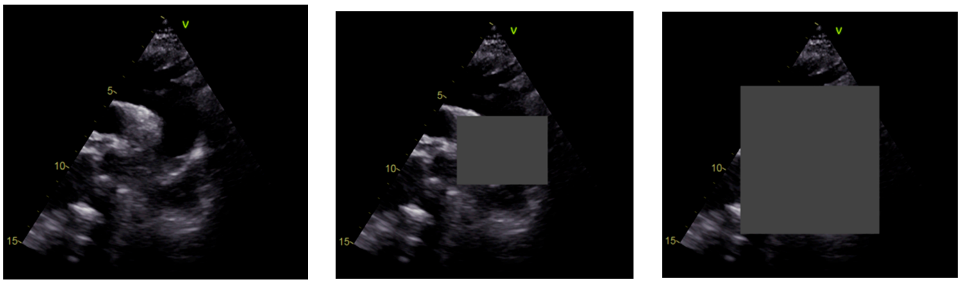

Supplementary Figure S3. occlusion experiment. From top to bottom, parasternal long-axis (PSLA), parasternal short-axis at the level great vessels (SB), parasternal short-axis at the level of papillary muscles or mitral (SM), apical four-chamber view (A4C), apical five-chamber view (A5C), apical two-chamber view (A2C), apical three-chamber view (A3C), subcostal four-chamber view (SUB4C), suprasternal notch Aortic arch (SUPAO). From left to right, original image, partially occluded and fully occluded image.

With the increase of the occlusion area, the predicted probability is decreasing rapidly, which indicates that our method can find the key areas, and is an effective model for cardiac view classification.

Supplementary Table S3 the predicted probability of occlusion experiment

Cardiac views	original image	Partial occlusion	Full occlusion
PSLA	0.9979	0.7007	0.0091
SB	0.9970	0.6383	0.0200
SM	1.0000	0.3029	0.00001
A4C	0.9999	0.2117	0.0001
A5C	0.9996	0.0388	0.0002
A2C	0.9998	0.2765	0.0001
A3C	0.9912	0.0006	0.0000
SUB4C	1.0000	0.1796	0.0000
SUPAO	1.0000	0.2967	0.0002
